# Photoplethysmography-Derived Flow-Mediated Dilation as a Non-Invasive Marker of Endothelial Function in Erectile Dysfunction

**DOI:** 10.3390/diagnostics16172710

**Published:** 2026-08-25

**Authors:** Murat Buyukaksu, Aslinur Sircan-Kucuksayan, Murat Ucar, Murat Canpolat

**Affiliations:** 1 Department of Biophysics, Medical Faculty, Akdeniz University, Antalya 07070, Türkiye; canpolat@akdeniz.edu.tr; 2 Department of Biophysics, Medical Faculty, Alanya Alaaddin Keykubat University, Antalya 07425, Türkiye; aslinursircan@gmail.com; 3 Department of Urology, Medical Faculty, Alanya Alaaddin Keykubat University, Antalya 07425, Türkiye; murat.ucar@alanya.edu.tr

**Keywords:** erectile dysfunction, endothelial function, photoplethysmography, flow-mediated dilation, vascular dysfunction

## Abstract

**Background**: Endothelial dysfunction plays a central role in the pathophysiology of erectile dysfunction (ED) and reflects systemic vascular impairment. Photoplethysmography-derived flow-mediated dilation (PPG-FMD) has emerged as a novel, non-invasive approach for assessing endothelial function. This study aimed to investigate the utility of PPG-FMD as a marker of systemic endothelial function in patients with ED without overt coronary artery disease. **Methods**: In this observational cross-sectional comparative study, patients with vascular ED (*n* = 24), non-vascular ED (*n* = 18), and healthy controls (*n* = 30) were evaluated. Endothelial function was assessed using PPG-FMD. Key parameters, including dilation index, time to maximum dilation, area under the curve, and dilation index slope, were analyzed and compared across groups. **Results**: PPG-FMD parameters indicated significantly impaired endothelial function in patients with vascular ED compared to both non-vascular ED patients and controls (*p* < 0.01). Dilation index and area under the curve were significantly reduced in the vascular ED group. **Conclusions**: These preliminary findings support the presence of systemic endothelial dysfunction in vascular ED and suggest that PPG-FMD–derived parameters may serve as a non-invasive indicator of endothelial function. Further studies incorporating larger cohorts and validation against established endothelial assessment methods are required to determine its clinical applicability.

## 1. Introduction

Erectile dysfunction (ED) is the inability to achieve or maintain an erection sufficient for sexual satisfaction. It is a significant health concern that affects millions of men and their partners worldwide [1]. While ED itself poses substantial quality-of-life issues, its strong association with cardiovascular diseases (CVD) underscores its broader clinical significance. Recent studies indicate that endothelial dysfunction is a key pathological mechanism underlying this relationship. Since the endothelium and nitric oxide (NO) play central roles in both erectile function and cardiovascular regulation, it is unsurprising that endothelial dysfunction contributes to the pathophysiology of both ED and CVD.

Erection is a complex process involving vascular, neurological, and hormonal pathways. Any disruption in this intricate system can result in ED. Among its various etiologies, vascular causes are the most prevalent, accounting for approximately 60% of cases [2]. Other contributing factors include neurological disorders, hormonal imbalances, psychological conditions, unhealthy lifestyle habits, medication side effects, and chronic diseases [3]. The prevalence of ED varies across studies, but it is estimated to affect 30–50% of men over 40 years old [2,4]. Furthermore, ED is not uncommon in younger men; one study reported that one in four ED patients is under the age of 40 [5].

ED is increasingly recognized as a precursor to various comorbid conditions, particularly CVD. It has been linked to hypertension, diabetes, metabolic syndrome, obesity, and chronic obstructive pulmonary disease [6]. This suggests that ED is not merely a localized dysfunction but a marker of systemic vascular health [7]. Given the crucial role of endothelial function in vascular homeostasis, its assessment in ED patients can provide valuable insights into both erectile and cardiovascular health.

Endothelial function is typically evaluated using Flow-Mediated Dilation (FMD), which is considered the gold standard for non-invasive endothelial function assessment [8]. This technique involves transient occlusion of the brachial artery followed by Doppler ultrasound measurement of the NO-mediated vasodilation response. However, despite its extensive use, FMD has significant limitations, including the need for expensive equipment, specialized software, and an experienced operator, making it less feasible for routine clinical practice.

Given these challenges, alternative methods have been developed to improve the practicality and accessibility of endothelial function assessment. One such method is Peripheral Arterial Tonometry (PAT), which utilizes the Endo-PAT device (Itamar Medical, Caesarea, Israel) [9]. PAT has been widely used for cardiovascular risk stratification and prognostication in coronary artery disease and other conditions [10,11,12,13].

More recently, Photoplethysmography Flow-Mediated Dilation (PPG-FMD) has emerged as a promising alternative. Compared to FMD and PAT, PPG-FMD is more accessible, operator-independent, and cost-effective. While still a relatively new technique, it has shown strong correlation with the gold standard USG-FMD method in different patient populations [9,14]. Its applications have been explored in various clinical contexts, including gestational hypertension [15] and coronary artery disease [14,16], with promising results.

Despite its increasing clinical use, PPG-FMD has not yet been systematically evaluated in ED patients. The present study aimed to assess endothelial function using the PPG-FMD method in patients diagnosed with ED who do not have coronary artery disease (CAD). Additionally, the study aimed to evaluate the effectiveness of PPG-FMD in differentiating between vascular and non-vascular causes of ED. Given its preliminary nature and limited sample size, this study should be regarded as a feasibility investigation intended to inform larger confirmatory studies.

## 2. Materials and Methods

### 2.1. Study Population and Design

The study was conducted at the Urology Outpatient Clinic of Alanya Alaaddin Keykubat University Training and Research Hospital. The study was approved by the Ethics Committee of Akdeniz University, Faculty of Medicine (protocol code: 39; date of approval: 11 January 2017). Patients presenting to the outpatient clinic who volunteered to participate were evaluated by an experienced urologist, diagnosed, and subsequently enrolled in the study. All participants were male. The present study is a clinic-based observational cross-sectional comparative study involving three groups. Patients with known coronary artery disease (CAD) were excluded from the study.

The differentiation between vascular and non-vascular ED was based on comprehensive clinical evaluation by an experienced urologist, taking into account the patient’s IIEF score, age, cardiovascular risk factors and comorbidities (e.g., diabetes, hypertension, dyslipidemia), the mode of symptom onset, and overall clinical history. A gradual, progressive onset in the presence of vascular risk factors was considered suggestive of vascular etiology, whereas an abrupt or situational onset in the absence of such factors was considered suggestive of a non-vascular etiology. Penile Doppler ultrasonography was not performed; this limitation is addressed in Section 4.

### 2.2. Measures

Erectile function of all participants was evaluated using the self-reported International Index of Erectile Function (IIEF) under the supervision of a urologist. Current biochemical analysis results were collected from laboratory records. Subsequently, participants were directed to a separate room within the outpatient clinic and informed about the study protocol. Following a 15-min rest period, demographic data were collected, anthropometric measurements were taken, and endothelial function was assessed.

**Figure 1:**
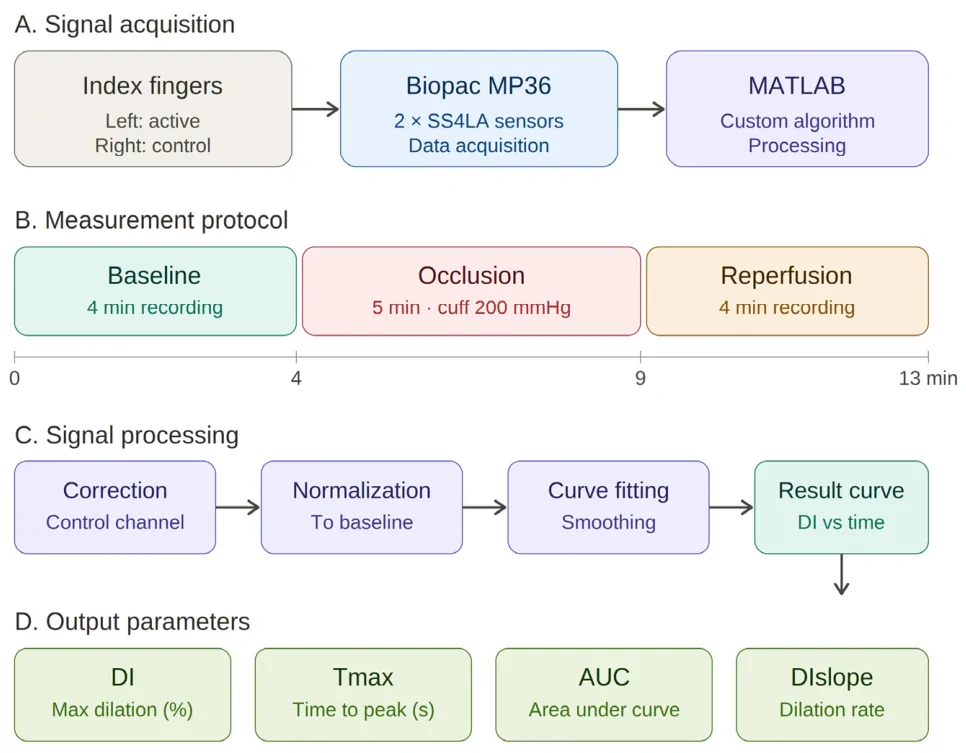
Overview of the PPG-FMD measurement and analysis workflow. (**A**) PPG signals were recorded from the index finger of each hand (left: active, right: control) using a Biopac MP36 module with two SS4LA sensors and processed in MATLAB. (**B**) Protocol: 4-min baseline, 5-min cuff occlusion at 200 mmHg, and 4-min reperfusion. (**C**) Signal processing: correction, normalization, and curve fitting to generate the result curve. (**D**) Derived parameters: dilation index (DI), time to maximum dilation (Tmax), area under the curve (AUC), and dilation index slope (DIslope). PPG-FMD: photoplethysmography-derived flow-mediated dilation.

The PPG-FMD method was employed for the measurement of endothelial function. Briefly, PPG-FMD is a non-invasive approach that measures the microvascular dilation response in the upper extremity during reactive hyperemia, following the standard FMD occlusion protocol (Figure 1).

Two photoplethysmography signals were acquired simultaneously using a Biopac MP36 data acquisition module (Biopac Systems, CA, USA) and two Biopac SS4LA photoplethysmography sensors placed on the index finger of both hands. The left-hand signal served as the active channel, on which occlusion was applied, while the right-hand signal served as a control channel used to correct for systemic vasoactive effects. After recording a baseline signal for 4 min, a cuff placed on the left upper arm was inflated to 200 mmHg and maintained for 5 min to achieve arterial occlusion. The cuff was then released, and the pulse signal was recorded for a further 4 min during the reactive hyperemia period.

**Figure 2:**
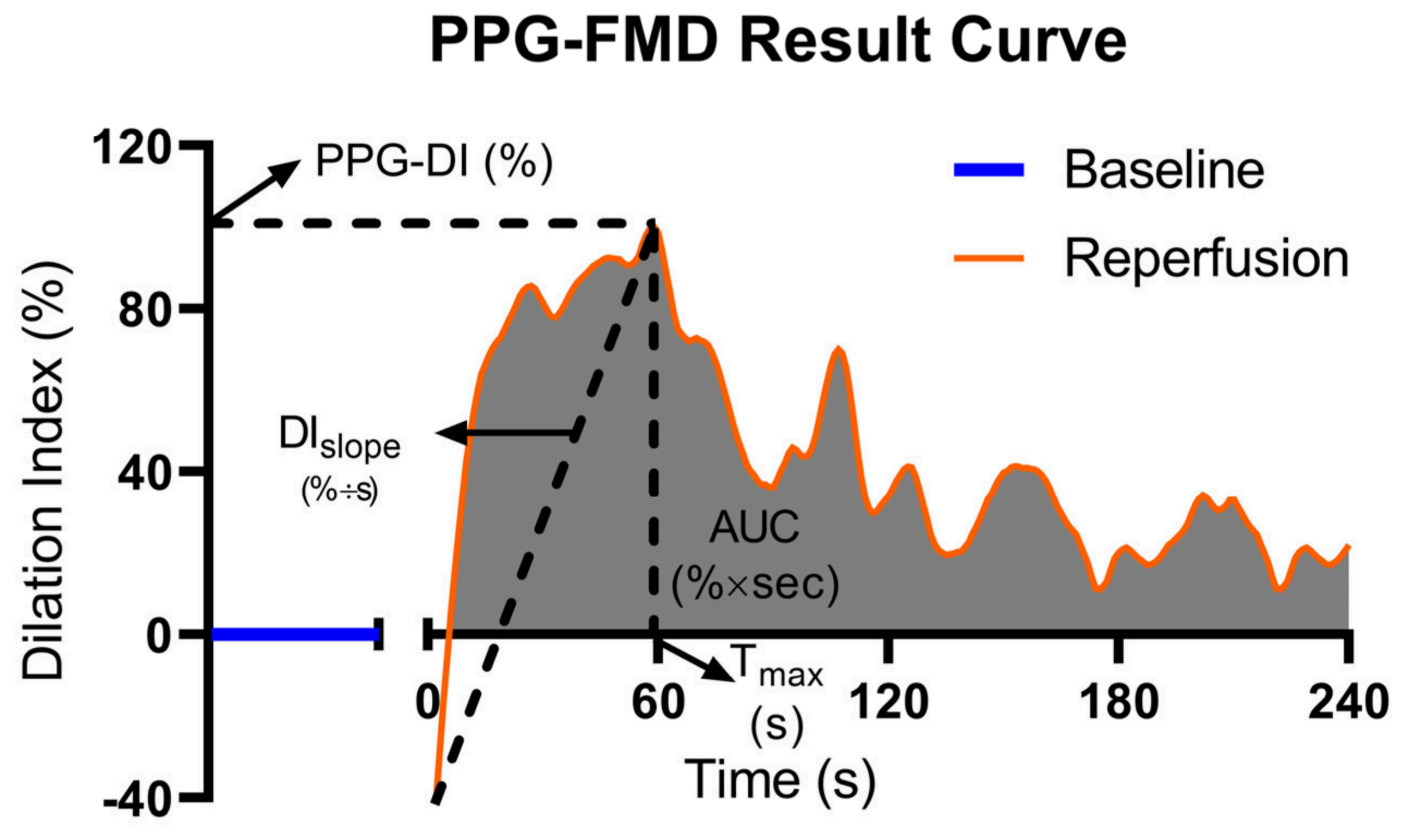
An illustrative DI over time and an explanatory representation of the calculated outcome parameters on the curve. The zero point on the time axis represents the moment when the occlusion ends and reperfusion begins. PPG-DI: maximum dilation index; DIslope: the slope of the line drawn from the onset of reperfusion to the point of maximum dilation index on the curve; Tmax: the time elapsed from the beginning of reperfusion to reach maximum response: AUC: the area under the curve of the portion of the curve above zero.

The recorded signals were transferred to the MATLAB environment (MathWorks, MA, USA), where all signal processing was performed using a custom-developed algorithm. A continuous, element-wise correction approach was applied to the active-channel signal using the control-channel signal to eliminate systemic vasoactive influences, followed by normalization to the pre-occlusion baseline. Curve fitting was then applied to obtain a smooth, continuous PPG-FMD result curve. The maximum value of this curve was defined as the Dilation Index (DI), representing the maximum percentage increase in pulse wave amplitude during the post-occlusion period. Physiologically, the DI corresponds to the maximum dilation response mediated by post-occlusive blood flow and shear stress on the vessel wall (Figure 2).

After determining the maximum dilation response as the Dilation Index, the time elapsed from the beginning of reperfusion to reach this response (in seconds) was recorded as Tmax, and the area under the curve (AUC) of the portion of the curve above zero was calculated and recorded. Finally, the average slope of the curve from the beginning of reperfusion to the maximum dilation response was calculated and recorded as DIslope. Full mathematical details of the continuous correction method have been described previously [17].

### 2.3. Data Analysis

Statistical analyses were performed using GraphPad Prism version 8.4.2. The normality of data distribution was assessed using the Shapiro–Wilk test. For comparisons across groups, one-way analysis of variance (ANOVA) with Tukey’s post-hoc test was applied for normally distributed continuous variables, and the chi-square test was used for categorical variables. Non-normally distributed continuous variables were compared using the Kruskal–Wallis test. A *p*-value of less than 0.05 was considered statistically significant. All continuous variables are expressed as mean ± standard deviation (SD), and categorical variables are expressed as number (percentage).

As no prior data on PPG-FMD in ED patients were available, the expected effect size was estimated from previous studies assessing endothelial function in ED using ultrasonographic FMD. Based on the difference in FMD values reported by Kaya et al. between ED patients and controls [18], and assuming a standard deviation consistent with previous FMD studies, a large effect size (Cohen’s d ≈ 1.4–1.8) was anticipated for the primary endothelial function comparison. Under these assumptions, a minimum of approximately 8–11 participants per group would provide 80% power at a two-sided significance level of 0.05. The sample sizes in the present study (vascular ED, *n* = 24; non-vascular ED, *n* = 18; controls, *n* = 30) therefore exceeded the minimum required for the primary comparisons, although power for the secondary parameters (Tmax and DIslope), which showed smaller between-group differences, was more limited.

Generative AI tools (ClaudeSonnet 5.0, Anthropic, PBC) were used to assist with statistical computations, including the calculation of effect sizes, confidence intervals, and sample size estimation, as well as with language editing of the manuscript. All analyses were verified by the authors, and the authors take full responsibility for the accuracy and integrity of the data, results, and interpretation presented in this work.

## 3. Results

### 3.1. Participants

The study included 24 patients with vascular ED, 18 patients with non-vascular ED, and 30 healthy controls. The demographic and clinical characteristics of the study groups are summarized in Table 1.

Significant differences were observed among the groups in terms of IIEF scores (*p* < 0.01). Pairwise comparisons revealed that both the vascular and non-vascular ED groups had significantly lower IIEF scores than the control group.

The Framingham Risk Score (FRS) was highest in the vascular ED group (7.7 ± 3.4), lowest in the non-vascular ED group (2.5 ± 4.2), and intermediate in the control group (5.6 ± 4.9). The difference among groups was statistically significant (*p* < 0.01), and pairwise comparisons showed that only the vascular ED vs. non-vascular ED difference reached statistical significance.

### 3.2. Demographics and Laboratory Values

The demographic characteristics and laboratory values of the study groups are presented in Table 1 and Table 2, respectively. Age, BMI, waist circumference, pulse rate, and blood pressure values were similar among the groups (*p* > 0.05 for all).

In the biochemical analyses (Table 2), fasting glucose, hemoglobin, HbA1c, HDL, and LDL levels differed significantly among the groups (*p* = 0.03, *p* = 0.02, *p* = 0.02, *p* < 0.01, and *p* = 0.01, respectively). LDL levels were highest in the vascular ED group, whereas HDL levels were lowest. Additionally, the non-vascular ED group exhibited significantly lower hemoglobin levels compared to the control group.

### 3.3. Endothelial Function Assessment

**Figure 3:**
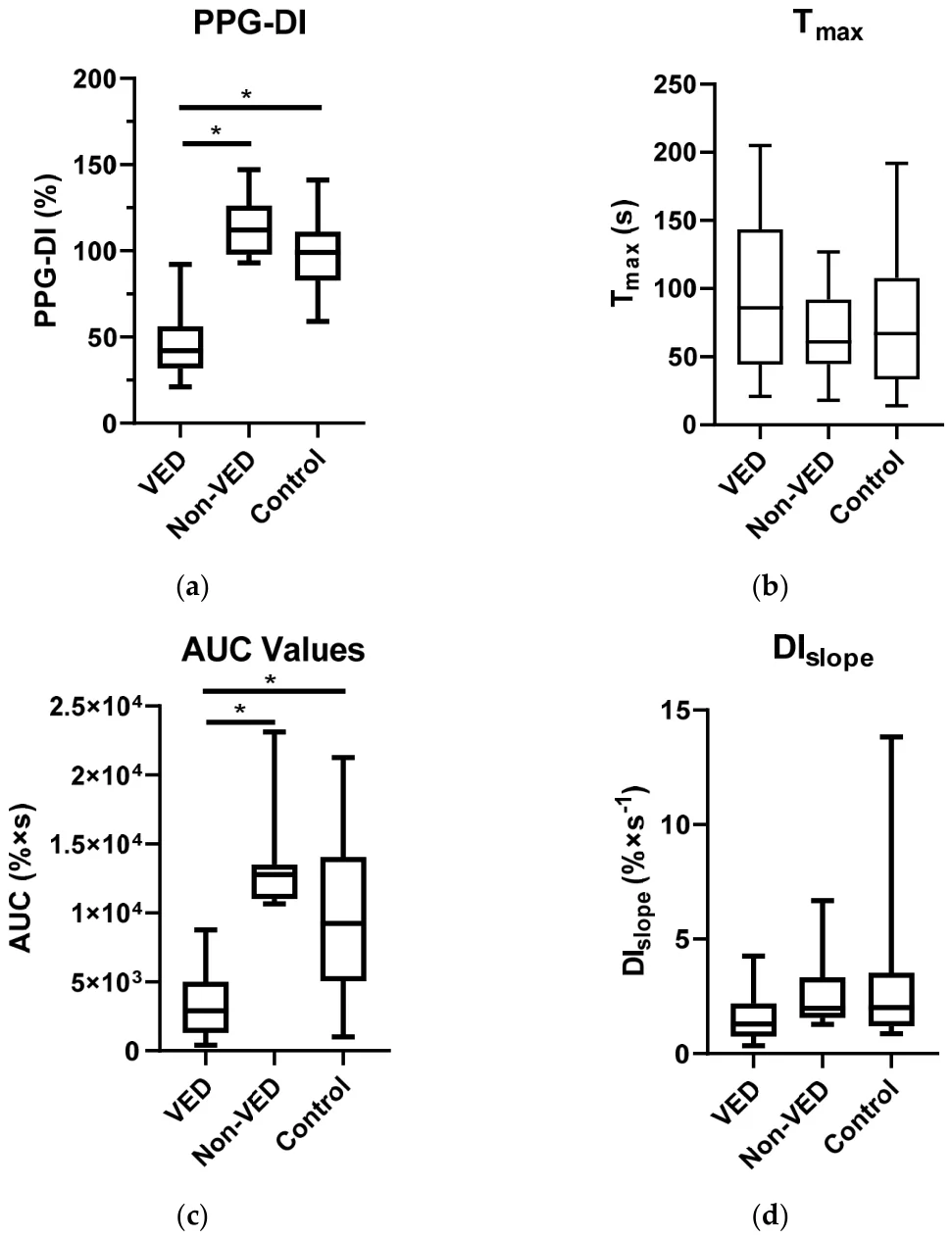
Endothelial function results of the study groups, including (**a**) dilation index, (**b**) Tmax values, (**c**) AUC values, and (**d**) DIslope values. Boxes represent the interquartile range (Q1–Q3), the horizontal line within each box indicates the median, and whiskers extend from the minimum to the maximum value. According to ANOVA, differences in the dilation index and AUC values were statistically significant (*p* < 0.01 for both). Pairwise comparisons are indicated in the figure (* *p* < 0.01).

Systemic endothelial function was assessed using the PPG-FMD method. Vascular ED patients exhibited significantly lower endothelial function compared to both the non-vascular ED and control groups (Figure 3a, *p* < 0.01), whereas no significant difference was observed between the non-vascular ED and control groups.

The mean PPG-Dilation Index (PPG-DI) values were 47.1% ± 20.6 in the vascular ED group, 113.6% ± 22.5 in the non-vascular ED group, and 97.7% ± 24.6 in the control group. Pairwise comparisons confirmed significantly lower PPG-DI in the vascular ED group compared to controls (*p* < 0.01; Cohen’s d = 2.21, 95% CI [38.54, 62.66]) and compared to the non-vascular ED group (*p* < 0.01; Cohen’s d = 3.11, 95% CI [53.25, 79.75]).

### 3.4. Additional Endothelial Function Parameters

In addition to PPG-DI, several other endothelial function parameters were analyzed (Figure 3). The Tmax parameter, representing the time required to reach maximum dilation, was 92.5 ± 58.3 s in the vascular ED group, 67.0 ± 43.3 s in the non-vascular ED group, and 74.4 ± 52.6 s in the control group. Although the vascular ED group exhibited a longer time to reach maximum dilation, the difference among groups was not statistically significant (*p* = 0.30; 95% CI [−11.87, 48.07] vs. controls).

The area under the curve (AUC), which reflects the magnitude and duration of the dilation response, was significantly lower in the vascular ED group (3329% × s ± 2396) compared to both the non-vascular ED (13,342% × s ± 4883) and control groups (9830% × s ± 6173) (*p* < 0.01; Cohen’s d = 1.33, 95% CI [4093, 8909] vs. controls; Cohen’s d = 2.73, 95% CI [7562, 12,464] vs. non-vascular ED).

The DIslope parameter, indicating the rate of vasodilation during the reperfusion period, was 1.68% × s^−1^ ± 1.32 in the vascular ED group, 2.84% × s^−1^ ± 3.01 in the non-vascular ED group, and 3.02% × s^−1^ ± 3.51 in the control group. Although vascular ED patients had a lower slope value, suggesting a slower dilation response, the difference was not statistically significant (*p* = 0.16; 95% CI [−2.70, 0.02] vs. controls).

**Figure 4:**
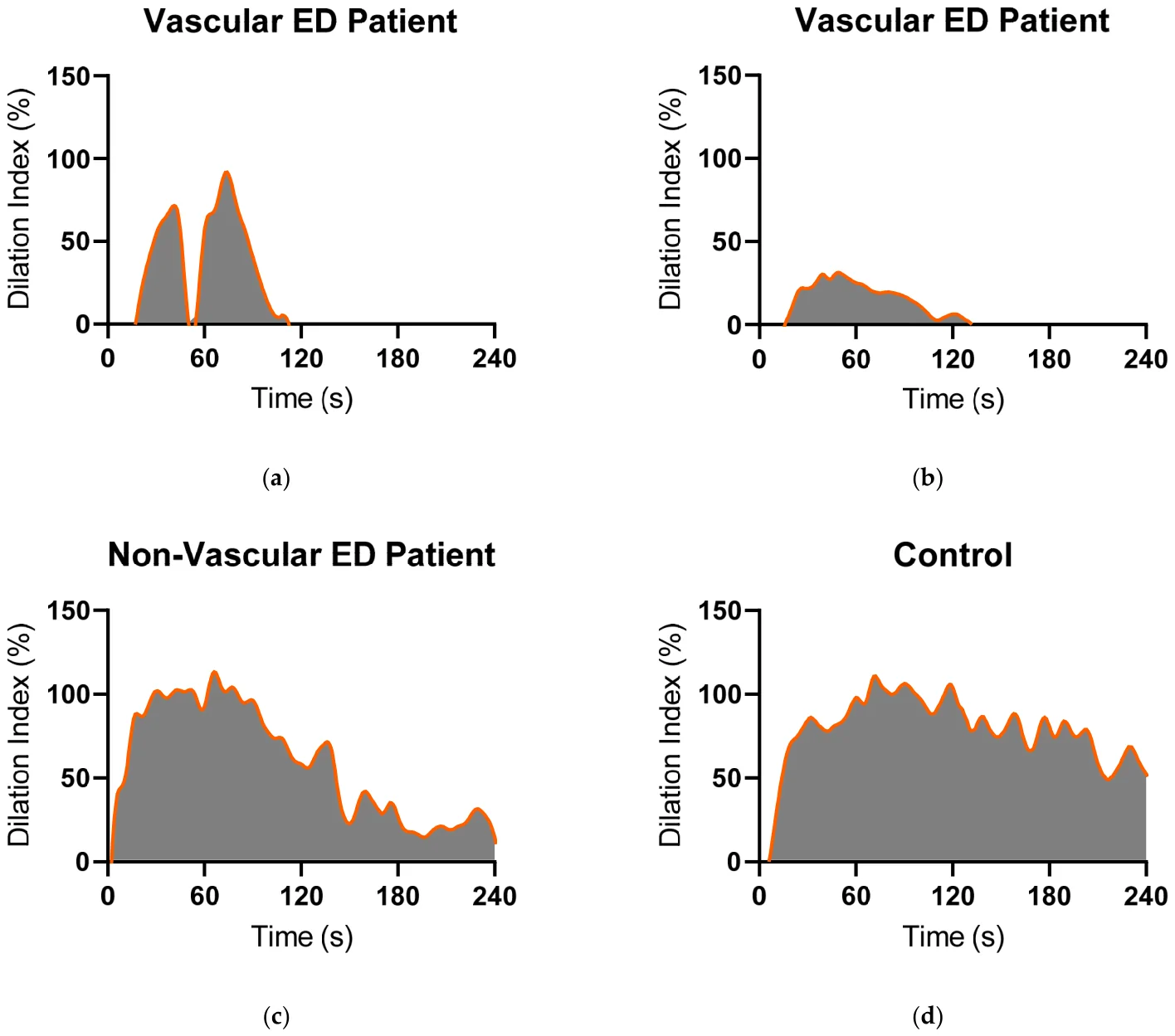
ED: Erectile dysfunction. Representative PPG-FMD result curves from individual participants: (**a**) a vascular ED patient, (**b**) another vascular ED patient, (**c**) a non-vascular ED patient, and (**d**) a control participant. The graphs illustrate qualitative differences in vasodilation responses across the groups. ED: erectile dysfunction.

Figure 4 presents PPG-FMD result curves from individual participants. Vascular ED patients (Figure 4a,b) exhibited delayed and weaker vasodilation responses compared to non-vascular ED and control participants (Figure 4c,d). In non-vascular ED and control groups, dilation occurred rapidly after reperfusion onset, with a sustained positive dilation response. In contrast, vascular ED patients showed a delayed transition to positive dilation, and the vasodilation response was markedly shorter in duration.

**Figure 5:**
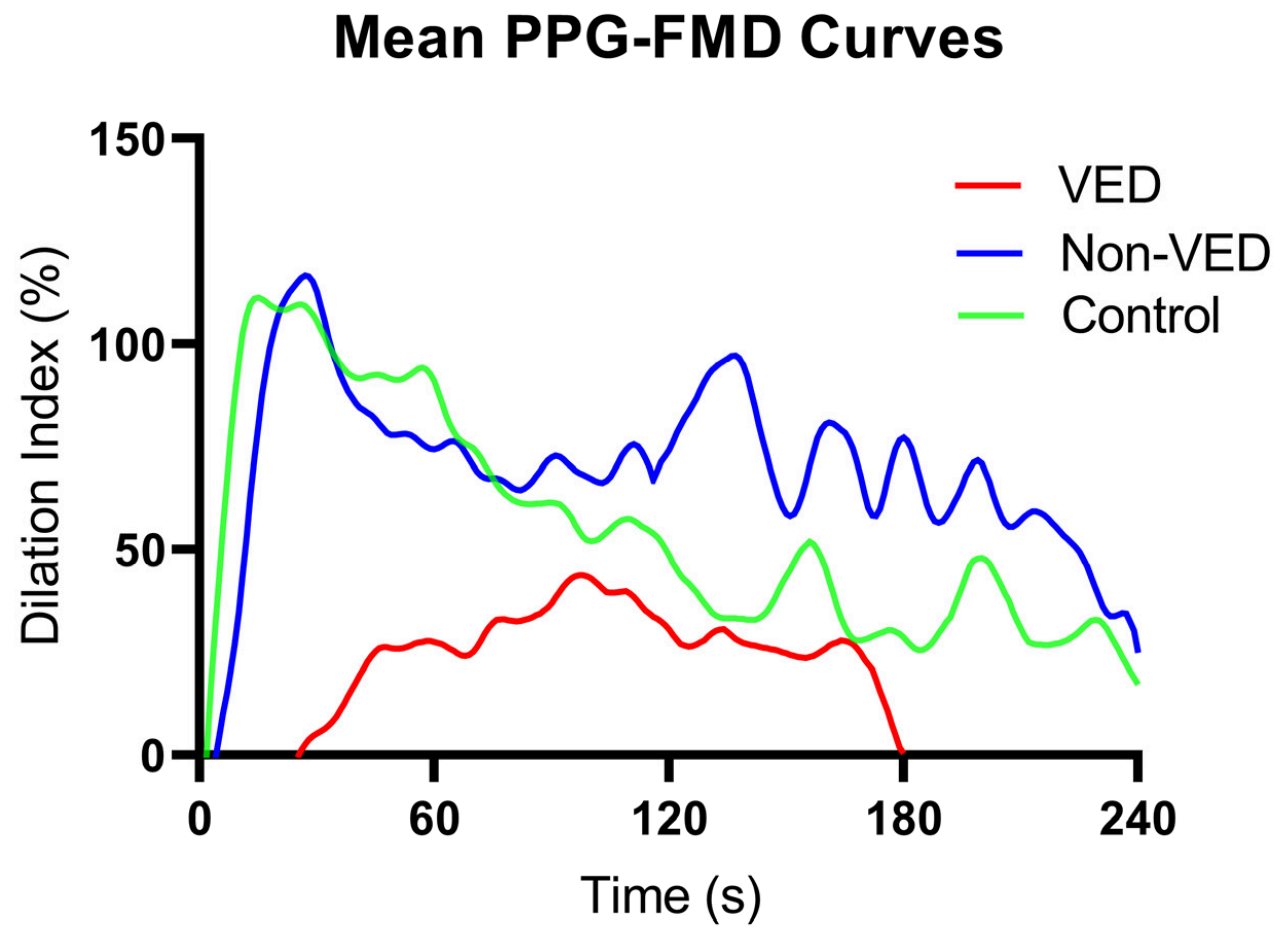
ED: Erectile dysfunction. Mean PPG-FMD curves of five randomly selected participants from each study group. The vascular ED (VED) group demonstrates a markedly attenuated and shorter-lasting vasodilation response compared to the non-vascular ED (Non-VED) and control groups. VED: vascular erectile dysfunction; non-VED: non-vascular erectile dysfunction; PPG-FMD: photoplethysmography-derived flow-mediated dilation.

Figure 5 displays the average PPG-FMD result curves for five randomly selected participants from each study group. The vascular ED group demonstrated a slower and reduced vasodilation response, whereas the non-vascular ED and control groups exhibited a more rapid and prolonged dilation pattern. The vascular ED curve dropped below baseline after three minutes, whereas in the other groups, it remained positive beyond four minutes, further emphasizing differences in the vasodilation response.

## 4. Discussion

Our findings align with the well-established relationship between erectile dysfunction (ED) and cardiovascular disease (CVD), further emphasizing the role of endothelial dysfunction as a shared pathological mechanism. Given that ED and CVD share common risk factors such as aging, smoking, diabetes, and hypertension, endothelial impairment in ED patients may serve as an early marker of systemic vascular dysfunction. The artery size hypothesis suggests that the smaller penile arteries are more susceptible to vascular narrowing before larger arteries, such as coronary vessels, exhibit symptoms. This may explain why ED often precedes major cardiovascular events, highlighting the importance of early endothelial function assessment in these patients.

Previous studies have shown that approximately two-thirds of men diagnosed with coronary artery disease (CAD) experienced ED prior to CAD symptoms [19]. Additionally, younger ED patients appear to have a higher predisposition to future cardiovascular disease compared to older individuals with ED [20]. These findings underscore the necessity of early screening and risk assessment in ED patients, particularly those with a vascular etiology, as endothelial dysfunction may be a critical predictor of future cardiovascular events.

Endothelial function plays a fundamental role in vascular homeostasis, primarily through the release of nitric oxide (NO), which mediates vasodilation in response to increased blood flow demands. Reduced NO bioavailability leads to a diminished and shorter-lasting vasodilation response, contributing to both erectile and cardiovascular dysfunction. Given its key role in vascular health, endothelial dysfunction is increasingly recognized as a major contributor to various vascular diseases. Although endothelial function measurement is not yet a routine clinical tool, accumulating evidence supports its use in assessing disease progression and prognosis.

In our study, PPG-FMD successfully detected endothelial dysfunction in vascular ED patients, showing significantly lower endothelial function compared to the non-vascular ED and control groups. These results reinforce the idea that systemic endothelial impairment is a hallmark of vascular ED and suggest that PPG-FMD may serve as a useful tool in differentiating between vascular and non-vascular ED etiologies.

Assessing endothelial function in ED patients provides two key benefits. First, it helps differentiate between vascular and non-vascular ED, allowing for more precise diagnostic and treatment strategies. Second, it enables the early identification of cardiovascular risk factors, facilitating timely intervention and risk modification to prevent future cardiovascular events. Given the strong link between ED, endothelial dysfunction, and cardiovascular disease, incorporating endothelial function assessments into routine evaluation could provide significant diagnostic and prognostic advantages.

Several studies have investigated endothelial function in ED patients, primarily using USG-FMD and PAT methods. Kaya et al. found that ED patients had significantly lower USG-FMD results than controls (6.0% vs. 12.3%), reinforcing the role of endothelial dysfunction in ED [18]. Similarly, Yavuzgil et al. demonstrated a clear distinction in endothelial function between ED patients, individuals with cardiovascular risk factors but no ED, and healthy controls (3.2%, 6.0%, and 10.2%, respectively) [21]. These findings suggest that measuring endothelial function in ED patients may provide insights into undiagnosed vascular disease and cardiovascular risk.

Studies utilizing PAT measurements in ED patients have yielded inconsistent results. While some studies found significant differences between ED and control groups [22,23], others, such as Mehta et al., reported no significant difference between general ED and post-prostatectomy ED patients (RHI: 1.97 vs. 2.08, *p* = 0.07) [24]. Similarly, Wu et al. observed a positive correlation between systemic and local penile endothelial function (r = 0.640, *p* < 0.001), suggesting that systemic endothelial assessments could be used to evaluate both ED and cardiovascular risk [25].

Our study is the first to utilize PPG-FMD for systemic endothelial function assessment in ED patients. Unlike PAT, which relies on pneumatic finger sensors, PPG-FMD detects vascular volume changes using LED and photodiode technology, providing a continuous, real-time assessment of endothelial responses. This may explain why PPG-FMD detected significant differences between vascular and non-vascular ED groups, whereas PAT-based studies have shown inconsistent results.

A major advantage of PPG-FMD is its ability to provide continuous measurement of dilation responses during each cardiac cycle. Unlike traditional FMD, which captures dilation at discrete time points, PPG-FMD offers real-time tracking of vascular responses, allowing for a more precise determination of peak dilation. Additionally, PPG-FMD does not require ECG synchronization, simplifying the assessment process and enhancing accuracy.

Beyond a single maximum dilation value, PPG-FMD provides multiple vascular parameters, including rate of dilation, time to peak dilation (Tmax), and total dilation response over time (AUC). Endothelial dysfunction is a multifaceted process, involving reduced NO synthesis, diminished NO sensitivity, and increased PDE-mediated NO degradation [26]. These mechanisms not only affect the magnitude of vasodilation but also its timing and duration. Given this complexity, PPG-FMD’s ability to analyze multiple parameters makes it a promising tool for both research and clinical applications.

Taken together, these features position PPG-FMD as a promising alternative to conventional endothelial function measurement techniques. While our findings support its clinical potential, further studies with larger cohorts and direct comparisons to USG-FMD and PAT are needed to establish its diagnostic accuracy and clinical applicability.

Several baseline differences between groups warrant careful consideration when interpreting our findings. The vascular ED group exhibited higher fasting glucose, HbA1c, and LDL levels, along with lower HDL levels, compared to the other groups. These cardiometabolic differences, together with variations in the prevalence of diabetes, smoking, and hypertension, are themselves established contributors to endothelial dysfunction. Consequently, the impaired endothelial function observed in the vascular ED group may partly reflect these coexisting risk factors rather than ED etiology alone. Similarly, medication use related to these conditions could have influenced endothelial responses. Because of the modest sample size, formal multivariable adjustment for these confounders was limited; therefore, the independent contribution of vascular ED to endothelial impairment cannot be fully isolated in this preliminary analysis. Larger studies with adequate statistical power are needed to disentangle these effects.

A further consideration relates to the age distribution across groups. Although the difference in age did not reach statistical significance (*p* = 0.14), the non-vascular ED group was younger on average than the vascular ED and control groups. Since age is a major determinant of the Framingham Risk Score (FRS), this discrepancy may have contributed to the notably lower FRS observed in the non-vascular ED group. As such, the between-group FRS comparison should be interpreted with caution, as it may partly reflect the underlying age difference rather than a true divergence in cardiovascular risk profile.

This study has several limitations. The small sample size and exclusion of ED patients with CAD may limit the generalizability of our findings. Additionally, the cross-sectional design prevents establishing a causal relationship between endothelial dysfunction and ED. Selection from a single outpatient clinic introduces potential selection bias, and the absence of Penile Doppler Ultrasonography may have affected the differentiation between vascular and non-vascular ED. Finally, uncontrolled factors such as medication use and lifestyle variables could have influenced endothelial function outcomes.

## 5. Conclusions

This preliminary study demonstrated that vascular ED patients exhibit significantly impaired endothelial function as assessed by PPG-FMD, reinforcing the role of endothelial dysfunction in its pathophysiology. In contrast, non-vascular ED patients had endothelial function comparable to controls, suggesting that psychogenic, neurogenic, or hormonal etiologies may predominate in this group. However, these findings should be interpreted in light of the modest sample size and baseline differences between groups.

These findings highlight the potential utility of PPG-FMD as a non-invasive tool for endothelial function assessment in ED evaluation. Future research should focus on validating PPG-FMD against established methods in larger, adequately powered cohorts with multivariable adjustment for confounding factors, and exploring its clinical utility in both ED diagnosis and cardiovascular risk stratification.

## Figures and Tables

**Table 1:** Characteristics of the study groups and *p* values of the comparisons.

	Vascular ED (*n* = 24)	Non-Vascular ED (*n* = 18)	Control (*n* = 30)	*p*
Age (year)	59.2 ± 7.3	49.3 ± 16.1	57.7 ± 15.9	0.14
BMI (kg/m^2^)	27.5 ± 3.9	26.2 ± 4.7	26.1 ± 3.3	0.31
Waist Cir. (cm)	100 ± 9.8	100 ± 14.4	96 ± 10.4	0.28
Pulse (bpm)	73 ± 14.7	70 ± 13.6	68 ± 18.6	0.45
SpO2 (%)	97.8 ± 1.5	99 ± 0.8	98 ± 1.1	0.26
SBP (mmHg)	137 ± 19.6	127 ± 12.7	133 ± 16.4	0.27
DBP (mmHg)	84 ± 9.8	81 ± 3.8	83 ± 8.8	0.66
Smoking (%)	9 (45)	4 (40)	8 (33)	0.73
Hypertension (%)	8 (40)	2 (20)	6 (25)	0.42
DM (%)	7 (35)	0 (0)	5 (21)	0.09
FRS	7.7 ± 3.4	2.5 ± 4.2	5.6 ± 4.9	**<0.01**
IIEF	11 ± 5	12 ± 4	28 ± 2	**<0.01**

Data were presented as mean ± SD. BMI: body mass index, bpm: beats per minute, SBP: systolic blood pressure, DBP: diastolic blood pressure, DM: diabetes mellitus, FRS: Framingham Risk Score, IIEF: international index of erectile function. Bold values indicate statistical significance (*p* < 0.05).

**Table 2:** Laboratory results of the study groups and *p* values of the comparisons.

	Vascular ED (*n* = 24)	Non-Vascular ED (*n* = 18)	Control (*n* = 30)	*p*
Fasting glucose (mg/dL)	117 ± 36.7	95 ± 11.0	101 ± 19.2	**0.03**
Creatinine (mg/dL)	0.9 ± 0.1	0.8 ± 0.1	0.9 ± 0.1	0.05
Hemoglobin (g/dL)	14 ± 1.0	13 ± 2.1	14.1 ± 1.1	**0.02**
HbA1c (%)	6 ± 1.0	5.5 ± 0.4	5.3 ± 1.1	**0.02**
Total cholesterol (mg/dL)	193 ± 16.2	181 ± 27.6	184 ± 17.0	0.13
HDL (mg/dL)	43 ± 7.8	48 ± 5.5	51 ± 7.1	**<0.01**
LDL (mg/dL)	123 ± 17.6	105 ± 23.8	108 ± 17.5	**<0.01**
Triglyceride (mg/dL)	135 ± 40.2	138 ± 55.2	127 ± 25.2	0.60

Data were presented as mean ± SD. HDL: high-density lipoprotein, LDL: low-density lipoprotein. Bold values indicate statistical significance (*p* < 0.05).

## Data Availability

The data presented in this study are available upon reasonable request from the corresponding author.

## Abbreviations

The following abbreviations are used in this manuscript:

CAD	Coronary Artery Disease
DI	Dilatation Index
DIslope	Dilatation Index Slope
FMD	Flow-Mediated Dilation
NO	Nitric Oxide
PAT	Peripheral Arterial Tonometry
PPG	Photoplethysmography
PPG-DI	Photoplethysmography Dilation Index
PPG-FMD	Photoplethysmography Flow-Mediated Dilation
Tmax	Time to Maximum Dilation
USG-FMD	Ultrasonographic Flow-Mediated Dilation
